# Breaking the Mucin Barrier: A New Affinity Chromatography-Mass Spectrometry Approach to Unveil Potential Cell Markers and Pathways Altered in Pseudomyxoma Peritonei

**DOI:** 10.1186/s12575-024-00239-0

**Published:** 2024-05-15

**Authors:** Antonio Romero-Ruiz, Melissa Granados-Rodríguez, Florina I. Bura, Francisca Valenzuela-Molina, Blanca Rufián-Andújar, Ana Martínez-López, Lidia Rodríguez-Ortiz, Rosa Ortega-Salas, María Torres-Martínez, Ana Moreno-Serrano, Justo Castaño, Carmen Michán, José Alhama, Mari C. Vázquez-Borrego, Álvaro Arjona-Sánchez

**Affiliations:** 1 Department of Biochemistry and Molecular Biology, University of Córdoba, Córdoba, Spain; 2 Maimonides Biomedical Research Institute of Córdoba, IMIBIC and University of Córdoba, Av. Menéndez Pidal, s/n, Córdoba, 14004 Spain; 3 Surgical Oncology Unit, Surgery Department, Reina Sofía University Hospital, Córdoba, Spain; 4 Pathology Unit, HURS, Córdoba, Spain; 5 Department of Cell Biology, Physiology, and Immunology, University of Córdoba, Córdoba, Spain; 6 CIBER Fisiopatología de La Obesidad y Nutrición, Córdoba, Spain

**Keywords:** Cancer, Mucin, Protein, Pseudomyxoma peritonei, MUC13

## Abstract

**Background:**

Pseudomyxoma peritonei (PMP) is a rare peritoneal mucinous carcinomatosis with largely unknown underlying molecular mechanisms. Cytoreductive surgery combined with hyperthermic intraperitoneal chemotherapy is the only therapeutic option; however, despite its use, recurrence with a fatal outcome is common. The lack of molecular characterisation of PMP and other mucinous tumours is mainly due to the physicochemical properties of mucin.

**Results:**

This manuscript describes the first protocol capable of breaking the mucin barrier and isolating proteins from mucinous tumours. Briefly, mucinous tumour samples were homogenised and subjected to liquid chromatography using two specific columns to reduce mainly glycoproteins, albumins and immunoglobulin G. The protein fractions were then subjected to mass spectrometry analysis and the proteomic profile obtained was analysed using various bioinformatic tools. Thus, we present here the first proteome analysed in PMP and identified a distinct mucin isoform profile in soft compared to hard mucin tumour tissues as well as key biological processes/pathways altered in mucinous tumours. Importantly, this protocol also allowed us to identify MUC13 as a potential tumour cell marker in PMP.

**Conclusions:**

In sum, our results demonstrate that this protein isolation protocol from mucin will have a high impact, allowing the oncology research community to more rapidly advance in the knowledge of PMP and other mucinous neoplasms, as well as develop new and effective therapeutic strategies.

**Supplementary Information:**

The online version contains supplementary material available at 10.1186/s12575-024-00239-0.

## Background

Pseudomyxoma peritonei (PMP) is a rare, malignant disease defined by a progressive, abundant, and multifocal accumulation of mucinous tumour tissue within the peritoneal cavity, with essentially no extraperitoneal growth nor distant metastases [1]. It has historically been considered a terminal condition for which debulking surgery or palliative treatments have been the only therapeutic options available. In recent years, several peer-reviewed studies have published results showing a survival benefit when treating PMP with cytoreductive surgery (CRS) combined with hyperthermic intraperitoneal chemotherapy (HIPEC) [1–4]. However, despite these therapeutic efforts, recurrence is common, with subsequent progression and death occurring in the absence of effective treatment.

Diagnosis of this entity is considered challenging, as the tumour may remain silent, mimic an episode of acute appendicitis, or be masked by abdominal disease. In addition, there are no specific tumour markers other than biopsy. For this reason, the different nomenclatures and classifications of the disease have always been debated. In this context, the most successful classification protocol is that of the Peritoneal Surface Oncology Group International (PSOGI), which has recently been validated and includes three subtypes of PMP: i) low-grade mucinous carcinoma (LG-PMP), ii) high-grade mucinous carcinoma (HG-PMP), and iii) PMP with the presence of signet ring cells (SRC-PMP) [5, 6]. Acellular mucin was excluded from this classification. This protocol has been validated and improved by our group [7], including the proposal of a new classification within the high-grade subtype based on the tumour marker Ki67 [8, 9].

The rapid development of personalised therapies for oncology patients has highlighted the importance of identifying distinct molecular profiles within the same histological tumour type. In this context, the search for personalised therapeutic solutions has made the molecular classification of tumours a critical step in clinical decision making. To date, only a few molecular studies of PMP cases have been published, all of which have been summarised in a recent narrative review by Lund-Andersen et al. [10]. Although some of the genes and proteins described in these studies have been proposed as biomarkers, their usefulness is still limited, and none have been validated as a potential therapeutic target. Furthermore, to our knowledge, there are no studies describing the protein profile of this mucinous tumour or any other peritoneal mucinous carcinomatosis, most likely because the high concentration of glycoproteins, together with the physical and chemical junctions between the mucins, interfere with any currently available protein isolation protocol [11].

Overall, mucins are a family of highly glycosylated proteins, also known as glycoproteins, expressed in specialised epithelial cells of mucosal surfaces, including those of the respiratory, gastrointestinal, and urogenital tracts [12, 13]. Mucins are divided into two subtypes: membrane-associated and secreted. Membrane-associated mucins act as sensors of the extracellular medium and can activate intracellular signalling pathways when the mucus layer is disrupted. Secreted mucins provide a physical barrier for epithelial cells against both microorganisms and insoluble materials and maintain the local molecular environment [14]. Under normal conditions, mucin production and degradation follow a well-established metabolic turnover. However, in PMP, mucin is secreted and gradually deposited in the peritoneal cavity where it becomes difficult to break down and eliminate. Over time, mucin accumulates, causing the symptoms associated with PMP, and can even surround tumour cells and protect them from the host immune response or the effects of chemotherapeutic agents [12]. An important finding in several studies is the presence of non-mucin proteins in mucus, such as digestive enzymes, dietary proteins, immunoglobulins, albumins, and keratin, which interact directly with mucin proteins through strong non-covalent interactions and increase the viscosity of PMP secretions [12, 15, 16].

As in other rare diseases, both the low incidence of PMP (3.2 cases per one million people/year) and the lack of molecular studies focused on this condition make it difficult to obtain a detailed description of its pathophysiology and thus to develop efficient, novel diagnostic, prognostic, or therapeutic strategies [17–19]. To address this issue, this study provides a description of the first protocol available to isolate total protein from mucin obtained from soft and hard tumour samples, which will undoubtedly advance research in this field, as it allowed us to identify a potential tumour cell marker.

## Methods

### Experimental design and statistical rationale

Soft and hard mucin samples were obtained from patients diagnosed with PMP of appendiceal origin and treated at our centre, as not all mucin PMP samples have a similar texture, compactness and hardness [11]. From a prospective cohort of 29 patients, we selected 18 low-grade (LG-PMP) tumour samples, including 15 soft mucin and 11 hard mucin samples, and 11 high-grade (HG-PMP) tumour samples, including 8 soft mucin and 11 hard mucin samples. It is important to note that soft and hard mucin samples were included for most patients. Additionally, we obtained 16 control tissue samples, nine of which were appendiceal samples obtained from a prophylactic appendectomy performed for another medical condition, and seven non-tumorous colon tissue samples obtained from PMP patients during a CRS with HIPEC procedure. All samples were immediately sent to the Pathology Unit for histological examination by experienced pathologists to confirm the diagnosis. The remaining samples were then snap frozen and stored in the biobank until requested for analysis. Importantly, all tissue samples were snap frozen within 30 min of resection. The demographic and histological findings of these examinations are detailed in Table 1. The samples used in each experiment are indicated in the figure legends. All patients signed an informed consent form, and the study was approved by our local ethics committee (protocol code PI19/01603).

**Table 1:** Demographic and histological results of PMP patients

	***Non tumoral***	***Tumour***	
		**Low-grade**	**High-grade**
*PATIENTS, [n]*	16	18	11
*SOFT MUCIN, [n]*	-	15	8
*HARD MUCIN, [n]*	-	11	11
*AGE, [median (IQR)]*	53 (20–86)	57 (27–79)	58 (23–74)
*WOMEN, (%)*	52,9	61,1	63,6

*IQR* Interquartile range

Soft and hard mucin samples were included for most patients

All statistical analyses were performed using software Prism v.8.0 (GraphPad Software, La Jolla, CA, USA), except for PLS-DA analyses, which were performed using MetaboAnalyst v.5.0 (McGill University, Quebec, Canada). Volcano plots were generated using R language v.4.1.2. For proteomic results, Welch t-tests were used to assess the existence of statistical differences between groups. All data are presented as a mean ± standard error of the mean (SEM). Unless otherwise stated, one-way analysis of variance (ANOVA) followed by post-hoc Bonferroni tests was used. *P*-values less than 0.05 were considered significant. Asterisks (* *P* < 0.05, ** *P* < 0.01, *** *P* < 0.001) indicate statistically significant differences.

### Adapted method to isolate proteins from mucin (AMIPROM)

One gram of both soft and hard mucin samples, obtained from LG and HG-PMP tumours, and control samples were dissected into small fragments and homogenised using ultrasound. The samples were then filtered and passed through the chromatograph on a HiTrap Con A 4B column to specifically capture glycoproteins, polysaccharides, and glycolipids. Flow-throughs corresponding to the maximum absorbance peaks identified in the two-dimensional plot chromatogram were collected and precipitated for use in the next step. The collected fractions were centrifuged and filtered again and then passed through the chromatograph a second time using a HiTrap Albumin and IgG Depletion column to specifically capture albumins and immunoglobulin G (IgG) (see “Liquid chromatography and protein isolation” for details). As in the previous step, the flow-through fractions corresponding to the maximum absorbance peaks identified in the chromatogram were collected and precipitated in acetone for mass spectrometry (MS) analysis.

Finally, all protein samples were precipitated, quantified, and properly prepared (see “SWATH-MS protein quantification” for details) to perform a nano-liquid chromatography coupled to tandem mass spectrometry (nano-LC/MS–MS) using a sequential window acquisition of all theoretical mass spectra (SWATH-MS) data independent acquisition (DIA) approach for massive protein quantification.

### Liquid chromatography and protein isolation

As mentioned above, mucin samples (~ 1 g) were dissected into small fragments (1–3 mm) and homogenised in 3.5–4.5 ml of binding buffer (20 mM Tris, 500 mM NaCl, 1 mM MnCl_2_, and 1 mM CaCl_2_) using ultrasonic pulses. After centrifugation and filtration through a 0.22-μm filter, 3–4 ml of each sample was loaded into the Äkta Purifier (Cytiva, MA, USA), which was previously loaded with the Hitrap Con A 4B column (Cytiva, MA, USA). Glycoproteins, polysaccharides, and glycolipids were captured in the column and the remainder of the sample was collected in 0.4 ml fractions corresponding to the maximum absorbance peaks. All collected fractions were then precipitated with four volumes of cold acetone, centrifuged, and the pellets were resuspended in 1.5 ml of binding buffer (20 mM NaH_2_PO_4_ and 150 mM NaCl). All fractions were mixed in a single tube, centrifuged, and filtered. Next, 0.5 ml of this filtered extract was reintroduced into the Äkta Purifier, which had previously been equipped with the HiTrap Albumin and IgG depletion column (Cytiva, MA, USA). As in the previous step, IgG and albumins were captured on the column and the remainder of the sample was collected in 0.2-ml fractions corresponding to the maximum absorbance peaks. These fractions were then precipitated with four volumes of cold acetone and some of them were used for mass spectrometry analysis. The remaining fractions were centrifuged, and the pellets were resuspended in 100 μl of DIGE lysis buffer (0.1 mM urea, 1 M thiourea, 30 mM Tris, and 4% (w/v) 3-[(3-cholamidopropyl)-dimethylammonium]-1-propanesulfonate) for Western blot and ELISA measurements.

### SWATH-MS protein quantification

Proteomic analysis was performed at the SCSIE proteomics facility (University of Valencia). Purified protein extracts obtained by liquid chromatography were precipitated and dissolved in 100 μl of lysis buffer (EasyPep™ Mini MS Sample Prep Kit; Thermo Fisher Scientific). The samples were then quantified using the Qubit™ Protein Assay Kit (Thermo Fisher Scientific) and 20 μg of each protein extract was digested and purified using the EasyPep™ Mini MS Sample Prep Kit (Thermo Fisher Scientific) according to the manufacturer’s instructions. The samples were then dried and resuspended in 2% acetonitrile (ACN) and 0.1% trifluoroacetic acid (TFA) to a final concentration of 1 μg/μl.

The samples were analysed using a SWATH DIA approach for massive protein quantification:

First, an equal amount of all samples from each group was pooled and subjected to a shotgun analysis to build a peptide spectral library. Each pooled sample was analysed twice by data-dependent acquisition (DDA) nanoscale liquid chromatography, followed by tandem mass spectrometry (nano LC–MS/MS) runs using LC system Ekspert™ nanoLC425 (Eksigent, Dublin, CA, USA) coupled to a Triple TOF® 6600 + (Sciex, Redwood City, CA, USA) mass spectrometer system. Next, 3 µl of each peptide mixture sample was loaded by the nanoLC425 system onto a trap column (3 µm C18-CL, 350 μm × 0.5 mm; Eksigent) and desalted with 0.1% TFA at a flow rate of 5 µl/min for five minutes. The peptides were then eluted on an analytical column (3 µm C18-CL 120 Ᾰ, 0.075 × 150 mm; Eksigent) equilibrated with 5% ACN and 0.1% formic acid (FA). Peptide elution was performed using a 60 min linear gradient of 7% to 40% buffer B (buffer A: 0.1% FA diluted in water; buffer B: 0.1% FA diluted in ACN) at a flow rate of 300 nl/min.

Eluted peptides were ionised in an Optiflow < 1 uL Nano Source, applying 3.0 kV to the spray emitter at 175 ºC, and analysed in a data dependent mode. Survey MS1 scans were acquired from 350–1400 m/z for 250 ms. The quadrupole resolution was set to ‘LOW’ for MS2 experiments, which were acquired from 100–1500 m/z for 25 ms in ‘high sensitivity’ mode. The following switching criteria were used: charge: 2 + to 4 + ; minimum intensity; 250 counts per second (cps). Up to 100 ions were selected for fragmentation after each survey scan. Dynamic exclusion was set to 15 s. Rolling collision energy equations were set for all ions as + 2 ions, according to the following equation: $\left|\mathrm{CE}\right|=\left(0.049\right)\times\left(\mathrm m/\mathrm z\right)+\left(2\right)$.

For quantification, the Triple TOF® was operated as above but in SWATH mode with a 0.050 s TOF MS scan ranging between 350 m/z and 1250 m/z. In addition, 0.080 s product ion scans were acquired in 100 variable windows ranging between 400 m/z and 1250 m/z throughout the experiment.

Peptide and protein identification was performed using Protein Pilot v5.0 software (Sciex). The Paragon algorithm [20] was used to search the Swissprot_200601.fasta database with the following parameters: trypsin specificity, iodoacetamide (IAM) cysteine alkylation, and taxonomy restricted to *Homo sapiens*. The MS/MS spectra of the identified peptides were used to generate a spectral library for the SWATH peak extraction using the add-in for the PeakView v2.1 software (Sciex) MS/MSALL with SWATH Acquisition MicroApp v2.0 (Sciex). Peptides with a confidence score ≥ 95% as reported by the Protein Pilot database search were included in the spectral library.

The mass spectrometry proteomics data were deposited with the ProteomeXchange Consortium via the PRIDE [21] partner repository under the dataset identifier PXD037364.

### Prediction of protein interactions and pathway enrichment analysis

The differentially expressed proteins identified in the SWATH quantitative analysis were imported into the Metascape database [22], which was used to perform protein–protein interaction enrichment analyses and functional enrichment analyses. For protein–protein interaction enrichment analyses, Metascape applies a mature complex identification algorithm called Molecular Complex Detection (MCODE) to automatically extract protein complexes embedded in a large network [23]. For functional enrichment analysis, the minimun overlap number and enrichment factor were 1.5 and the *p*-value cut-off was 0.05.

### Western Blot

The protein concentration of each sample obtained by liquid chromatography was determined using the RC DC™ Protein Assay (Bio-Rad). The absorbance of the samples was read at a wavelength of 750 nm in a standard spectrophotometer (Beckman DU530). Briefly, 5 μg of the total protein sample was subjected to SDS-PAGE on 9% polyacrylamide gels, electrotransferred to polyvinylidene difluoride membranes (Millipore) and probed overnight at 4 °C in the presence of the appropriate primary antibody (anti-MUC13 [1/500; Abcam]). A horseradish peroxidase-conjugated secondary antibody (anti-rabbit [1/5000; Abcam]) was used for protein detection and a chemiluminescence ECL Western Blotting Substrate (Thermo Fisher Scientific) was applied. Protein levels were normalised using the total line normalisation (TLN) method, where the intensity value of the total protein present in each sample was calculated. In addition, densitometric analysis of the resulting protein bands was performed using Image J.

### ELISA measurements

Protein extracts obtained by liquid chromatography were also used to determine the presence of MUC13 using a commercial human ELISA kit (MUC13 [MBS507589; MyBiosource] according to the manufacturer´s instructions.

## Results

### Proteomic profile in Pseudomyxoma peritonei

As we mentioned above, molecular characterisation of PMP is scarce and there are only a few papers showing some mucin isoforms (mainly MUC2 and MUC5AC) analysed by Western blot [15, 16]. Importantly, the results of these experiments were highly smeared band patterns due to the high levels of mucin proteins and their interactions. Consequently, these protein extracts are unsuitable for high throughput proteomic analyses.

**Fig. 1:**
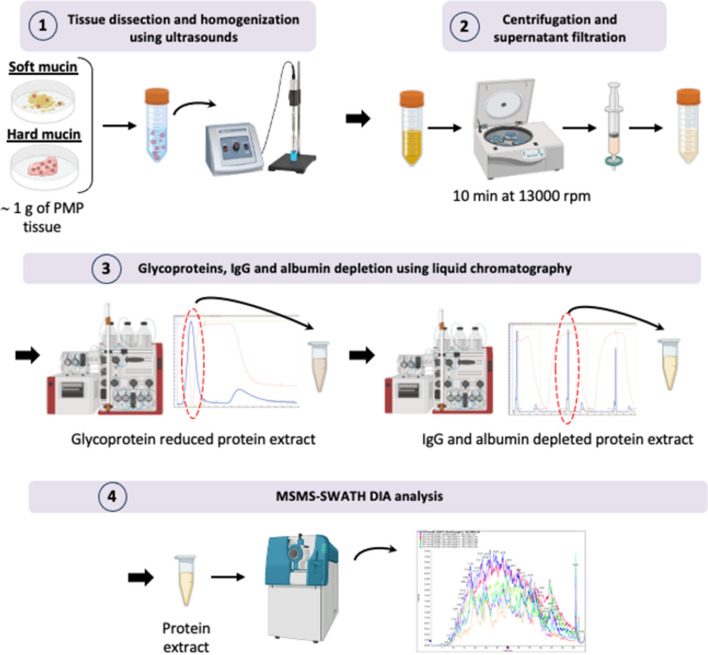
Schematic of the adapted method to isolate proteins from mucin. Adapted method to isolate proteins from mucin (AMIPROM): samples of soft and hard mucin obtained from LG-PMP and HG-PMP are cut into small pieces and homogenised using ultrasound. The homogenate is then centrifuged and the supernatant is collected and filtered. The homogenate is then subjected to liquid chromatography (LC) using two different columns to reduce the most abundant glycoproteins, IgGs, and albumin from the sample. The LC-derived protein extract is subjected to mass spectrometry analysis using an unbiased targeted proteomic approach with SWATH-MS

To overcome this problem, we developed the first method specifically adapted to mucin samples to extract and isolate total protein with a high quality and purity for their use in proteomic analyses (Fig. 1). Thus, soft and hard mucin samples from low and high-grade PMP tumours processed by this method, called *AMIPROM,* were used to determine the proteomic profile of PMP in order to identify intracellular pathways altered in PMP as well as potential tumour cell markers through the application of quantitative proteomics. Healthy samples from an appendectomy performed for an unrelated medical condition or normal colon tissue were used as controls due to the lack of appropriate non-tumour mucin samples (Table 1).

**Fig. 2:**
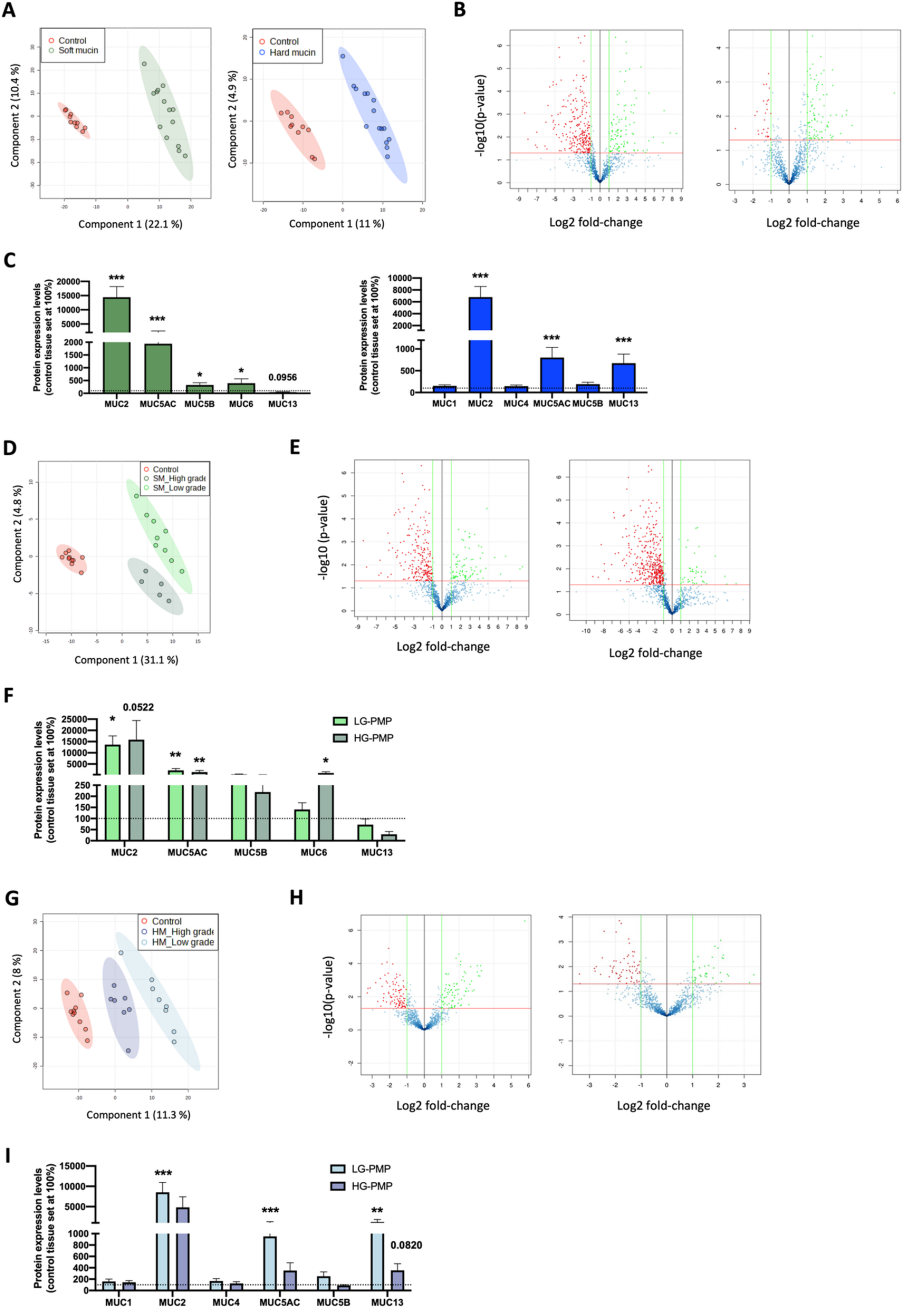
Proteomic analysis of soft and hard mucin obtained from LG-PMP and HG-PMP samples compared to control samples. **A** Partial least squares discriminant analysis (PLS-DA) of the proteome profile between soft (left; *n* = 14) and hard (right; *n* = 15) mucin samples and control tissues (*n* = 10). **B** Volcano plots showing Log2 Fold Change expression vs –log10 (*p*-value) of differentially expressed proteins with a *p*-value < 0.05 and an absolute Log2 Fold Change > 1 in the same sample set. Green colour indicates up-regulated proteins and red colour indicates down-regulated proteins. **C** Protein expression levels of mucin isoforms identified in soft mucin (green bars) and hard mucin (blue bars) mucin samples from PMP compared to control tissue (set to 100%; dashed line). **D**, **G** PLS-DA analysis of the proteome profile of low and high-grade soft mucin (SM) (D) and hard mucin (HM) (G) samples compared to control tissues. **E**, **H** Volcano plots showing Log2 Fold Change expression vs –log10 (p-value) of differentially expressed proteins with a *p*-value < 0.05 and an absolute Log2 Fold Change > 1 in low (left panel) and high-grade (right panel) soft mucin (E) and hard mucin (H) samples compared to control tissues. **F**, **I** Protein expression levels of identified mucin isoforms in low (light bars) and high-grade (dark bars) soft mucin (F) and hard mucin (I) samples compared to control tissue (set to 100%; dashed line). * *p* < 0.05 and, ** *p* < 0.01, *** *p* < 0.001

Nano-LC/MS–MS equipped with SWATH for label-free quantitative proteomics allowed us to generate the first proteome profile described in PMP. Considering all these proteins, a partial least squares discriminant analysis (PLS-DA) revealed a clear discrimination pattern between the proteomic profile of the soft mucin, hard mucin and control tissue samples (Fig. 2A). Furthermore, using a log2-fold change difference > 1 and a *p*-value < 0.05 to determine differentially expressed proteins compared to the control tissues, we identified 93 up-regulated and 243 down-regulated proteins in the soft mucin samples and 86 up-regulated and 27 down-regulated proteins in the hard mucin samples (Fig. 2B). Considering that mucins are the main proteins that characterise this entity, we then proceeded to identify the different mucin isoforms detected in the analysed PMP subtypes and detected a differential pattern of mucin isoforms between the soft and hard mucin samples (Fig. 2C). Specifically, we detected MUC2, MUC5AC, MUC5B, MUC6, and MUC13 in the soft mucin samples compared to the control tissues, with MUC2, MUC5AC, MUC5B, and MUC6 being significantly upregulated in these samples (Fig. 2C; left graph) and MUC1, MUC2, MUC4, MUC5AC, MUC5B, and MUC13 in the hard mucin samples, with MUC2, MUC5AC, and MUC13 being significantly upregulated in these samples (Fig. 2C; right graph). MUC2, although reduced by the glycoprotein affinity column, was the most highly expressed mucin isoform in all cases.

We then performed an analysis to compare LG and HG PMP soft mucin samples, which revealed that PLS-DA could perfectly separate both groups and clearly distinguish them from the control samples (Fig. 2D). Considering only differentially expressed proteins compared to the control tissue samples, volcano plots showed 72 up-regulated and 220 down-regulated proteins in the LG-PMP soft mucin samples (Fig. 2E-left panel) and 19 up-regulated and 380 down-regulated proteins in the high-grade soft mucin samples (Fig. 2E-right panel). As we mentioned above, although we found a different pattern of mucin isoforms between soft and hard mucin samples, no significant differences were found between the mucin isoforms identified in the LG and HG soft mucin samples (Fig. 2F). In the same line, PLS-DA performed on LG and HG hard mucin samples revealed a clear discrimination pattern between the overall proteomic profile of these samples and that of the control tissues (Fig. 2G). Notably, as shown by the volcano plots (Fig. 2H), the number of differentially expressed proteins was significantly higher for soft mucin than for hard mucin compared to the control tissues. Furthermore, the expression levels of MUC5AC and MUC13 were significantly upregulated in LG but not in HG hard mucin samples compared to control tissues. However, there were no statistical differences in their expression between LG and HG hard mucin samples (Fig. 2I).

Finally, we analysed the extracts captured in the two columns used during the protocol to evaluate how many proteins were depleted/reduced along with glycoproteins, albumins, and immunoglobulins. To do this, we performed an electrophoresis on the extracts from the columns and the major bands were excised and analysed by mass spectrometry (see Supplemental Methods). The results are shown in Figure S1, Table S1 and Supplemental Excel 1. The results were analysed using a cut-off of the 1% spectral count value of the most abundant protein identified for each column. Briefly, a total of 85 proteins were identified in the extracts captured on the HiTrap Con A 4B column, of which 82 were secreted proteins (including glycoproteins), 15 were cell membrane proteins and 7 were cytosolic proteins. It is important to note that most of the cell membrane and cytosolic proteins are also considered secreted proteins. For the HiTrap Albumin and IgG depletion column, a total of 16 proteins were identified, all of which were secreted proteins (including albumin and immunoglobulins) and 7 of which were cell membrane proteins. In addition, 10 out of 15 (66.7%) of the identified cell membrane proteins captured on the HiTrap Con A 4B column and all (100%) of the identified cell membrane proteins captured on the HiTrap Albumin and IgG depletion column were immunoglobulins, which were one of the targets to be depleted.

### Soft and hard mucin tissues are highly similar at functional level

**Fig. 3:**
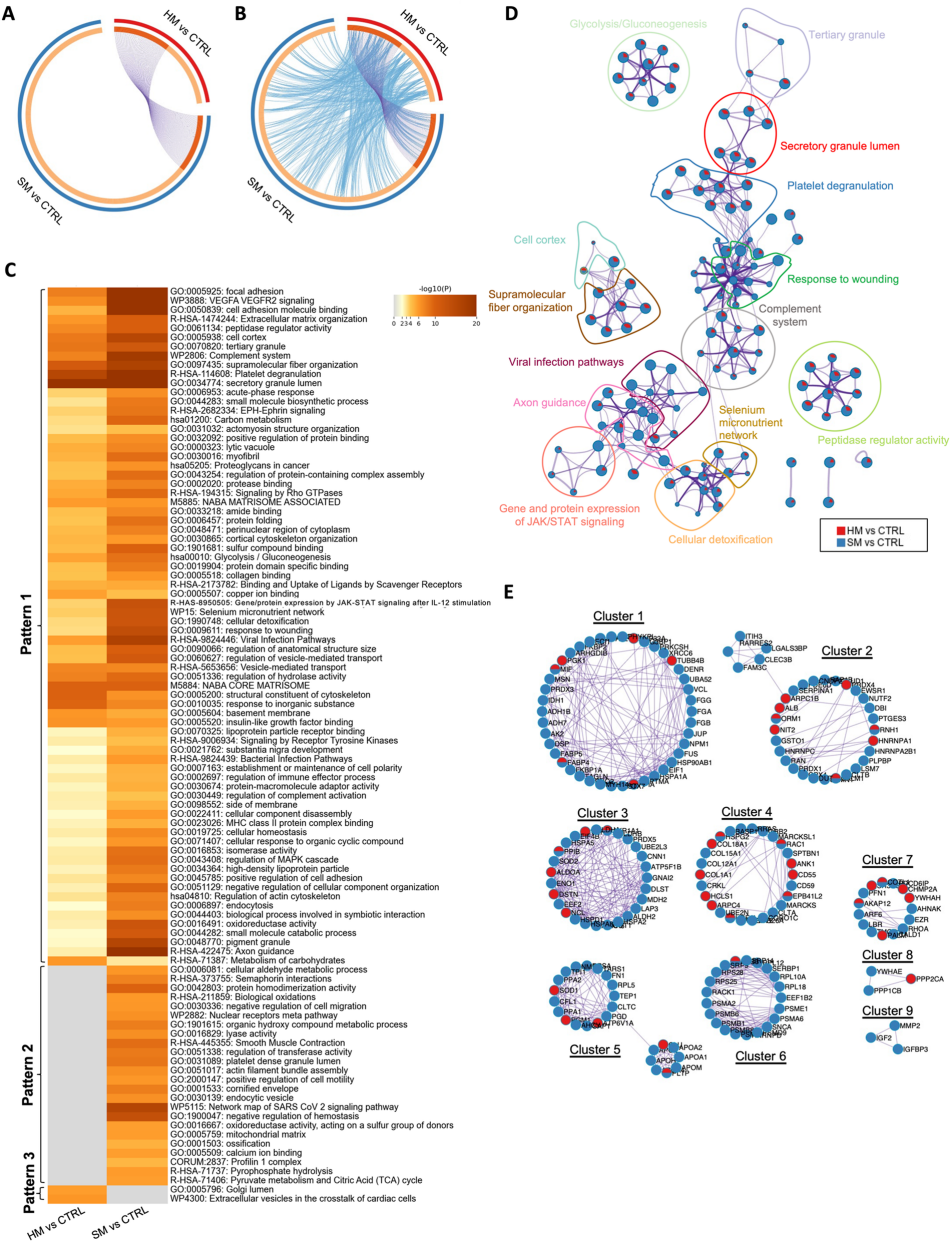
Visualisation of the functional enrichment meta-analysis based on two protein lists [soft mucin (SM) vs. control (CTRL) and hard mucin (HM) vs. control (CTRL)]. **A** Circos plot visualisation of the overlap between the protein lists (SM vs. CTRL and HM vs. CTRL). Each candidate protein is assigned to a point on the arc of the corresponding protein list(s). Proteins common to both lists are connected by purple curves. **B** Circos plot visualisation with blue curves connecting those candidate proteins that have different identities but share an enriched pathway/process, i.e. they represent the functional overlap between the protein lists. **C** Heatmap showing the top 100 enrichment clusters, one row per cluster, using a discrete colour scale to represent statistical significance. Grey colour indicates the lack of enrichment for that term in the corresponding gene list, light yellow colour indicates the boundary between significance and insignificance, deep yellow colour indicates a high degree of significance. **D** Enrichment network visualisation for results from the two protein lists, where nodes are represented by pie charts indicating their associations with each input list. Cluster labels have been added manually. The colour code represents the identities of the protein lists, where blue indicates SM vs. CTRL and red indicates HM vs. CTRL. **E** Visualisation of the PPI network and MCODE components identified from the combined protein list, where each node represents a protein with a pie chart encoding its origin. Colour codes for pie sectors represent a protein list

To explore the functional relevance of soft and hard mucin PMP tissues, all differentially expressed proteins found in soft and hard mucin compared to control tissues were analysed using the Metascape database. In this sense, we found an 84.2% of unique proteins in soft mucin compared to controls and 53.1% in hard mucin samples compared to controls, with only a small fraction of proteins shared between both comparisons, as illustrated by the Circos plot (Fig. 3A). Interestingly, although most of the differentially expressed proteins were different between the two comparisons, they shared a high number of enriched pathways and processes (Fig. 3B, C). Indeed, from the top 100 enriched terms included in the heatmap (where the colour scale represents statistical significance), most of them were significantly altered in both comparisons (pattern 1), with the terms “Golgi lumen” and “Extracellular vesicles in the crosstalk of cardiac cells” enriched exclusively in the comparison hard mucin vs. control (pattern 3) and the terms “Cellular aldehyde metabolic process”, “Biological oxidations”, “Negative regulation of cell migration”, among others, enriched exclusively in the comparison soft mucin vs. control (pattern 2) (Fig. 3C). In general, an elevated number of enriched processes were related with extracellular matrix (including “extracellular matrix organisation”, “Naba matrisome associated”, “Naba core associated”, “collagen binding”, “focal adhesion”, etc.), regulation of cytoskeleton (including “actomyosin structure organisation”, cortical cytoskeleton organisation”, “structural constituent of cytoskeleton”, etc.), metabolism (including “Glycolysis/Gluconeogenesis”, “Carbon metabolism”, “metabolism of carbohydrates”, “pyruvate metabolism and Citric Acid (TCA) cycle”, etc.), and signalling pathways highly related with cancer (including “VEGFA VEGFR2 signalling”, “EPH-Ephrin signalling”, “signalling by Rho GTPases”, “regulation of MAPK cascade”, etc.).

To facilitate the understanding of pathways/processes that are shared between the two comparisons, additional visualisations were developed. For example, an enrichment network visualisation including the results from both comparisons confirmed the same results as the heatmap, showing an overlap of biological processes by both soft and hard mucin tissues, as these proteins probably are likely to capture different parts of the same biological processes (Fig. 3D). Furthermore, a protein–protein interaction (PPI) network was also generated to elucidate common/selective functional clusters. In this sense, 9 different clusters were identified based on the MCODE algorithm, most of which were shared between both comparisons, and only cluster 9 (related to regulation of insulin-like growth factor transport and uptake) was specifically enriched in soft mucin vs. control. Cluster 6 (related to signalling by ROBO receptors and metabolism of amino acids) was also mainly enriched in soft mucin vs. control (Fig. 3E and Table S2).

### Low and high-grade soft mucin tissues share a high number of biological processes/pathways

**Fig. 4:**
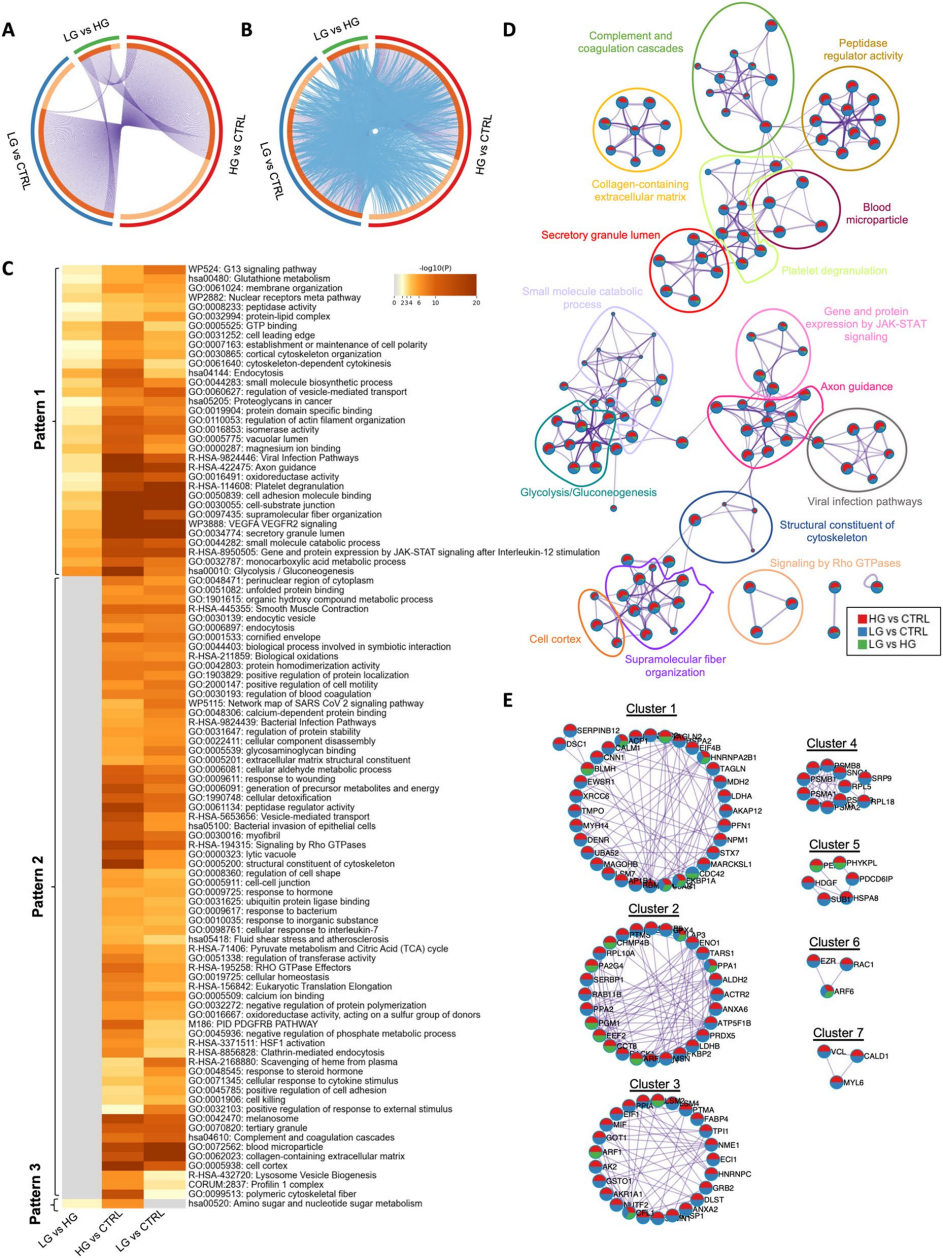
Visualisation of the functional enrichment meta-analysis based on three protein lists [LG vs. control (CTRL), HG vs. control (CTRL) and LG vs. HG)] in soft mucin samples compared to control tissues. **A** Circos plot visualising the overlap between the protein lists. Each candidate protein is assigned to a point on the arc of the corresponding protein list(s). Proteins common to both lists are connected by purple curves. **B** Circos plot visualisation with blue curves connecting those candidate proteins that have different identities but share an enriched pathway/process, i.e. they represent the functional overlap between protein lists. **C** Heatmap showing the top 100 enrichment clusters, one row per cluster, using a discrete colour scale to represent statistical significance. Grey colour indicates the lack of enrichment for that term in the corresponding gene list, light yellow colour indicates the boundary between significance and insignificance, deep yellow colour indicates a high degree of significance. **D** Enrichment network visualisation for results from the three protein lists, where nodes are represented by pie charts indicating their associations with each input list. Cluster labels have been added manually. Colour code represents the identities of protein lists, where blue indicates LG vs. CTRL, red indicates HG vs. CTRL and green indicates LG vs. HG. **E** Visualisation of the PPI network and MCODE components identified from the combined protein list, where each node represents a protein with a pie chart encoding its origin. Colour codes for pie sectors represent a protein list

Next, we wanted to explore the functional relevance of LG and HG soft mucin PMP tissues. Therefore, we compared all differentially expressed proteins found in LG and HG soft mucin compared to control tissues and in LG compared to HG using the Metascape database. In this case, the Circos plot showed an elevated number of differentially altered proteins shared between the three comparisons, with only 28.4% of unique proteins in LG vs. control, 40.6% of unique proteins in HG vs. control and 20.6% of unique proteins in LG vs. HG (Fig. 4A). Interestingly, 94% of the proteins detected in the LG vs. HG comparison were common to the HG vs. control protein list, suggesting that most of these proteins are specifically altered in HG. In terms of functional enrichment, an increased number of biological processes/pathways were found to be altered in both LG vs. control and HG vs. control, which was corroborated by comparing all differentially altered proteins in LG vs. HG, showing a high number of common enriched terms between LG and HG (grey colour; pattern 2) (Fig. 4B-C). Among these common terms, we found processes and pathways related to protein regulation (e.g. “unfolded protein binding”, “protein homodimerisation activity”, “positive regulation of protein localisation”, regulation of protein stability”, “negative regulation of protein polymerisation”, etc.), metabolism (e.g. “biological oxidations”, “cellular aldehyde metabolic process”, “generation of precursor metabolites and energy”, “pyruvate metabolism and citric acid (TCA) cycle”, etc.), and extracellular matrix (e.g. “glycosaminoglycan binding”, “extracellular matrix structural constituent”, “cell–cell junction”, “collagen-containing extracellular matrix”, etc.). In addition, we found some enriched terms that were differentially altered between LG and HG (pattern 1), which could be used to understand the differences between these two disease grades. Some of these terms were related to the regulation of the cytoskeleton (e.g. “establishment or maintenance of cell polarity”, “cortical cytoskeleton organisation”, “cytoskeleton-dependent cytokinesis”, “endocytosis”, “regulation of vesicle-mediated transport”, “regulation of actin-filament organisation”, etc.), signalling pathways highly related with cancer (e.g. “G13 signalling pathway”, “nuclear receptors meta pathway”, VEGFA VEGFR2 signalling”, and “gene and protein expression by JAK-STAT signalling after Interleukin-12 stimulation”), metabolism (e.g. “glycolysis/gluconeogenesis”, “monocarboxylic acid metabolic process”, “small molecule catabolic process”, “isomerase activity”, “peptidase activity”, etc.), and other important extracellular matrix-related pathways such as “Proteoglycans in cancer”. The pathway “amino sugar and nucleotide sugar metabolism” was also found to be altered in LG vs. HG and in HG vs. control, but not in LG vs. control, suggesting that this pathway might be specifically altered in HG-PMP samples (Pattern 3; Fig. 4C). Consistent with these results, the enrichment network visualisation showed the same overlapping pattern, with most of the enriched terms shared between HG vs. control (red) and LG vs. control (blue), and only a few of them also shared with the LG vs. HG comparison (green) (Fig. 4D). Furthermore, the PPI network revealed 7 different functional clusters, all of them related to the cytoskeleton, signalling pathways and metabolism, and all shared between the three protein lists (Fig. 4E; Table S3).

### Identification of functional enrichment terms specifically associated with LG or HG hard mucin tissues

**Fig. 5:**
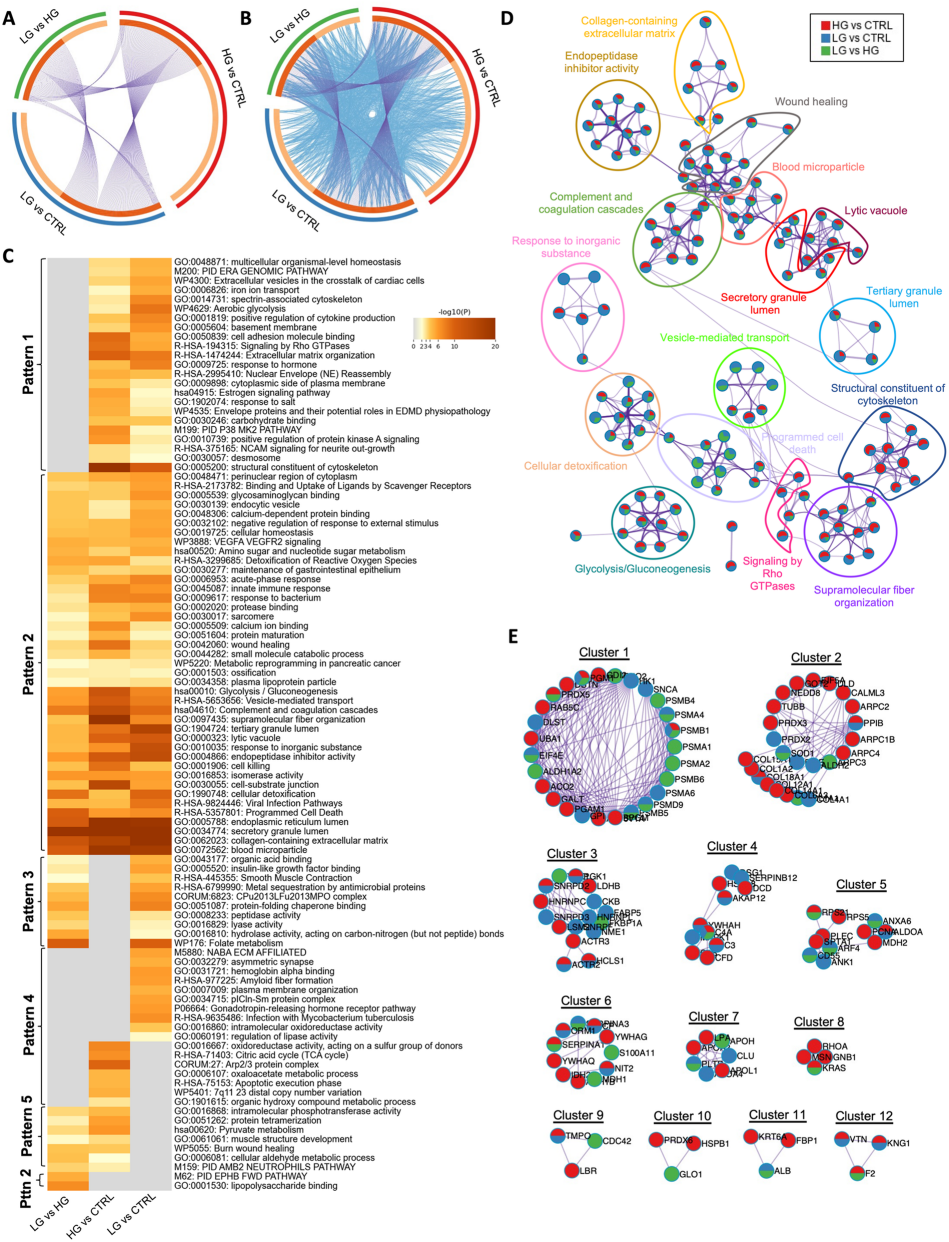
Visualisation of the functional enrichment meta-analysis based on three protein lists [LG vs. control (CTRL), HG vs. control (CTRL) and LG vs. HG] in hard mucin samples compared to control tissues. **A** Circos plot visualising the overlap between protein lists. Each candidate protein is assigned to a point on the arc of the corresponding protein list(s). Proteins common to both lists are connected by purple curves. **B** Circos plot visualisation with blue curves connecting those candidate proteins that have different identities but share an enriched pathway/process, i.e. they represent the functional overlap between protein lists. **C** Heatmap showing the top 100 enrichment clusters, one row per cluster, using a discrete colour scale to represent statistical significance. Grey colour indicates the lack of enrichment for that term in the corresponding gene list, light yellow colour indicates the boundary between significance and insignificance, deep yellow colour indicates a high degree of significance. **D** Enrichment network visualisation for results from the three protein lists, where nodes are represented by pie charts indicating their associations with each input list. Cluster labels have been added manually. Colour code represents the identities of protein lists, where blue indicates LG vs. CTRL, red indicates HG vs. CTRL and green indicates LG vs. HG. **E** Visualization of the PPI network and MCODE components identified from the combined protein list, where each node represents a protein with a pie chart encoding its origin. Colour codes for pie sectors represent a protein list

We then examined the functional relevance in LG and HG hard mucin tissues as we did above for soft mucin tissues. Circos plot analysis revealed a higher number of differentially altered proteins in the LG vs. HG comparison in hard mucin compared to the number of differentially altered proteins found in soft mucin (Figs. 4A and 5A). In general, the number of shared proteins between the three protein lists was lower in hard mucin tissues than in soft mucin tissues, with a higher number of unique proteins (48% of unique proteins in LG vs. control, 61.3% in HG vs. control and 36.9% in LG vs. HG) (Fig. 5A). The functional enrichment derived from these protein lists generated a heatmap with five different patterns according to the distribution of the enriched terms. Patterns 1 and 4 (grey colour) show all common enriched terms found between LG and HG, but differentially altered compared to control tissues. This is also illustrated in the Circos plot (Fig. 5B, C). Pattern 2 includes all enriched terms that are able to discriminate LG from HG hard mucin tissues. Among these terms we found processes/pathways related to metabolism (e.g. “amino sugar and nucleotide sugar metabolism”, “small molecule catabolic process”, “glycolysis/gluconeogenesis”, etc.), cellular homeostasis and detoxification (e.g. “detoxification of reactive oxygen species”, “cellular detoxification”, “programmed cell death” and immune response (e.g. “acute-phase response”, “innate immune response”, “complement and coagulation cascades”, etc.). In addition, patterns 3 and 5 revealed differentially enriched terms that are specifically associated with LG or HG hard mucin PMP tissues. For example, pattern 3 included important cellular activities such as “peptidase activity”, “lyase activity”, “hydrolase activity”, and also other processes such as “organic acid binding”, “insulin-like growth factor binding” and “protein-folding chaperone binding”, which may be specifically associated with LG hard mucin tissues, as they were altered in LG vs. HG and LG vs. control, but not in HG vs. control. In the same line, pattern 5 included important metabolic processes such as “pyruvate metabolism” and “cellular aldehyde metabolic process” and others such as “protein tetramerization” and “intramolecular phosphotransferase activity”, which could be associated with HG hard mucin tissues (Fig. 5C).

As before, to better understand the pathways/processes that are shared between the three comparisons, we generated the enrichment network visualisation and the PPI network. In the enrichment network, we found that most of the enriched terms were shared by the three protein lists, as they mainly represented patterns 1 and 2 from the heatmap. Nevertheless, processes such as “vesicle-mediated transport” and “programmed cell death” were mainly associated with LG vs. control and LG vs. HG, suggesting that these processes were mainly altered in LG-PMP tissues (Fig. 5D). Furthermore, the PPI network revealed 12 different functional clusters, all of them related to the proteasome, collagens, mRNA processing, complement activation and secretory granule lumen. Although no cluster was found to be specific to any protein list, clusters 2, 8 and 10 were mainly enriched in HG vs. control and LG vs. HG protein lists (Fig. 5E; Table S4).

### Validation of MUC13 alteration in soft and hard mucin samples of Pseudomyxoma peritonei

**Fig. 6:**
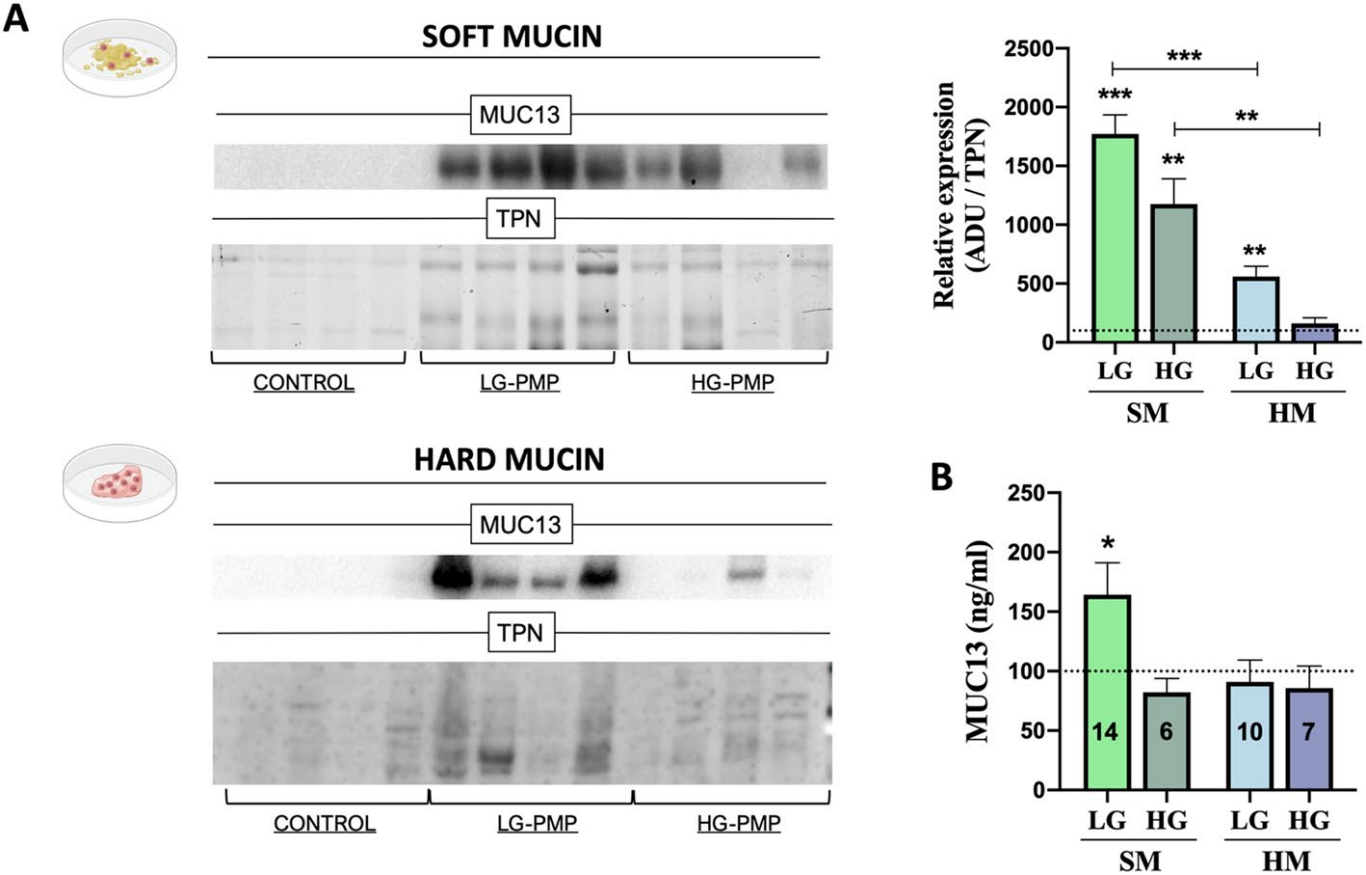
Validation of MUC13 expression levels in PMP. **A** Protein expression levels of MUC13 in soft mucin (SM – green bars) and hard mucin (HM – blue bars) [low (LG-PMP; *n* = 4) and high-grade (HG-PMP; *n* = 4)] compared to control tissues (*n* = 4; no tumoral appendix) evaluated by Western Blot. The arbitrary densitometric unit (ADU) for each protein was normalised by the Total Protein Normalisation (TPN) value. **B** Cohort validation of MUC13 by ELISA quantification SM and HM samples [low (LG-PMP) and high-grade (HG-PMP)] compared to control tissues (*n* = 16) (number of PMP samples analysed is indicated in the bars of the graph). One-way ANOVA analysis was performed for multiple comparisons (LG and HG-PMP vs Control). * *p* < 0.05 and *** *p* < 0.001

As we mentioned above, mucin isoforms are the main entity characterising this pathology. Interestingly, we observed a different mucin isoform pattern between soft and hard mucin tissues, with MUC13 being the only membrane-associated mucin found to be altered (Fig. 2), making it a potential candidate to be considered as a cellular tumour marker or cellular therapeutic target. For these reasons, MUC13 was quantified by Western blot and an enzyme-linked immunosorbent assay (ELISA) in soft and hard mucin samples obtained from patients with PMP (Fig. 6). In the Western blot analysis (Fig. 6A), MUC13 showed significantly higher expression in LG and HG soft mucin and in LG hard mucin tissues compared to control tissues, and was also found to be overexpressed in LG and HG soft mucin compared to hard mucin. In addition, MUC13 was quantified by ELISA in a larger cohort of PMP samples and was found to be overexpressed in LG soft mucin samples compared to control tissues (Fig. 6B). Importantly, MUC13 was not found in the depleted extracts (Supplemental Excel 1).

## Discussion

The aberrant production of mucin, the most common phenotype of PMP, has hampered the isolation of proteins from mucus-producing cells because their structure is intrinsically designed to avoid invasion by external agents, such as microorganisms or insoluble material [14]. In this study, we describe the first protocol designed to isolate proteins in this context and present the first proteomic profile of PMP. Bioinformatic analyses validated these pioneering data and identified a new potential tumour cell marker for PMP.

The main limitation in understanding the pathophysiology of rare diseases and finding therapeutic solutions for these conditions is the availability of samples. However, in the specific case of PMP, the lack of detailed and efficient protein isolation protocols that allow functional studies to be carried out is even more limiting. In this sense, all molecular studies published on PMP are based on histological data and expression analyses of genes already described in other cancers, mainly in colorectal and appendiceal neoplasms. The tumour-related genes *KRAS*, *GNAS*, *FAT4*, *TGFBR1*, *TP53*, and *SMAD3/4* may be mutated in this context, with *KRAS* and *GNAS* being the most frequently mutated and most studied genes in PMP [10]. However, there is still considerable controversy due to the lack of information on the functional implications of these transcriptional alterations [24–26]. Nevertheless, apart from a few studies on the expression and mutation of specific tumour-related genes, no analyses have been published that focus on the actual protein component involved in these tumours.

To overcome this handicap and to isolate proteins from PMP tumour samples, we developed the AMIPROM protocol, based on affinity liquid chromatography and the reduction of the most abundant mucus-forming glycoproteins, IgG, and albumins. In addition, AMIPROM allowed us to develop the first PMP protein library, which allowed us to perform a differential expression analysis by mass spectrometry DIA, revealing more than 300 deregulated proteins. Thus, for the first time, this protocol breaks the mucin barrier in PMP and allows access to the protein fraction in this rare tumour type, opening new perspectives for other more common mucinous tumours, such as mucinous colorectal cancer (CRC), which accounts for 10%—20% of CRC patients [27]. Furthermore, this protocol is a reliable option for the search of potential tumour cell membrane markers, as only secreted proteins were reduced along with the most abundant glycoproteins, albumin and immunoglobulins.

Current diagnostic and prognostic methods for PMP are based on histological data [6]. The development of a classification method based on molecular characterisation is mandatory in this scenario. The first PMP protein profile described in this work shows a great capacity to classify tumour and control samples of both soft and hard mucin, but it also allows us to distinguish between LG and HG soft and hard mucin PMP samples. At this point it is important to consider that PMP tissue samples have lower cellularity than the control tissues, mainly due to the mucinous component. Furthermore, Pillai et al. have provided evidence that hard mucin tissues have higher cellularity than soft mucin tissues [11, 28], and it is also described that HG PMP tumours have more abundant cellularity than LG PMP tumours [5]. Taking this information into account, all protein extracts were quantified and the same protein concentration was used in all cases for mass spectrometry analysis. To determine whether secreted proteins rather than intracellular proteins are responsible for the variations in the number of dysregulated proteins seen in soft mucin vs. control or hard mucin vs. control, an alternative experimental strategy should be used. For example, the mucinous component of the cells could be separated to extract the protein content independently, and mass spectrometry analysis could be performed on these extracts.

On the other hand, we wanted to elucidate the mechanisms (biological processes and signalling pathways) underlying this pathology. To do this, we used the Metascape database to perform functional enrichment and PPI networks. We paid special attention to all the altered proteins and regulatory networks that were uniquely enriched in the different groups of samples. Interestingly, the results derived from the analysis of the soft mucin vs. control and hard mucin vs. control comparisons (without distinction of histological grade) showed that although most of the altered proteins were different in each comparison, the terms associated with these alterations were very similar, suggesting that these proteins are probably different parts of the same biological processes and therefore that soft and hard mucin tissues are similar at the functional but not at the structural level. These results are complementary to those published by Pillai et al. who showed that soft, semi-hard and hard mucin samples had different textures and hardness, probably due to cell content, hydration, glucose, proteins, lipids, thiols and mucin distribution [11]. In addition, the enriched biological processes/pathways altered in these comparisons (mainly related to extracellular matrix, regulation of cytoskeleton, metabolism and signalling pathways highly related with cancer) confirmed the occurrence of tissue adaptation to promote malignant tumour progression [29].

Next, we wanted to elucidate the biological processes that might distinguish between LG and HG tumours in soft and hard mucin samples. In this sense, we found that there is a higher number of altered proteins shared between LG and HG in soft mucin than in hard mucin samples. In line with this, while there are a high number of common processes between LG and HG in soft mucin, we were able to identify processes/pathways in hard mucin that could specifically help to understand and better distinguish between LG and HG PMP. Thus, important cellular activities (e.g.“peptidase activity”, “lyase activity”, “hydrolase activity”, etc.) were specifically associated with LG hard mucin tissues and metabolic processes such as “pyruvate metabolism” and “cellular aldehyde metabolic process” and other processes were specifically associated with HG hard mucin tissues. In line with this notion, these cellular activities and tumour-associated metabolic deregulation have been described at different stages of carcinogenesis, and it is now clear that these alterations involve all stages of the cell-metabolite interaction, increasing the tumour’s ability to acquire nutrients, determining how the nutrients are preferentially allocated to specific metabolic pathways, contributing to cell cycle alteration and modifying cell differentiation [29, 30]. Furthermore, we found specific enriched terms related to extracellular matrix, cytoskeleton, metabolism and immune system, among others, that were differentially altered between LG and HG tumours in soft and hard mucin samples and could help to better understand this pathology, although we couldn’t specifically associate them with LG or HG. The fact that PMP is a tumour associated with advanced age often implies the presence of prior alterations in the extracellular matrix and the immune system of the patients affected by this disease. In this context, detailed descriptions have been published of how cellular and molecular changes in non-cancerous cells during ageing may contribute to a tumour-permissive microenvironment. These changes include biophysical alterations in the extracellular matrix, changes in secreted factors, and changes in the immune system [31], which certainly warrant further investigation in PMP.

Finally, our results revealed a different mucin isoform profile between soft and hard mucin, with MUC13 being the only membrane-associated mucin found to be altered. Individual quantification of MUC13 in a larger number of samples confirmed its aberrant synthesis in PMP tissues. MUC13 is a transmembrane protein expressed in mucin-producing epithelial cells whose main function is to activate an inflammatory response when an external agent damages the mucosal layer [32]. There are currently no data on the expression of MUC13 in serous or mesothelial cells. All this information, together with the fact that MUC13 has been reported to be overexpressed in several cancers, detected in the serum of patients with various cancers, and associated with increased cancer plasticity and the development of chemoresistance [33, 34], makes it a novel and interesting membrane protein target for detecting the presence of residual tumour cells after CRS-HIPEC treatment.

In conclusion, in this work we have broken the mucin barrier by developing AMIPROM, the first protocol available to isolate proteins from mucin, which has been the main obstacle to molecular studies of this rare cancer. In addition, we provide the first proteomic profile of PMP described to date, providing novel information to characterise and identify the pathways altered in this tumour. Furthermore, our differential protein expression analysis followed by bioinformatic approaches has, for the first time, revealed the biological and molecular processes involved in PMP genesis and identified a potential tumour cell marker. Overall, we believe that our study provides essential, original information to facilitate rapid advances in the knowledge of PMP pathogenesis, which is undoubtedly the first step towards the development of new and effective therapeutic tools to treat mucinous neoplasms.

### Supplementary Information

Supplementary Material 1.

Supplementary Material 2.

## Data Availability

The datasets used and analysed in this study are available upon reasonable request from the corresponding authors (b72rorua@uco.es and carmen.vazquez@imibic.org). In addition, the mass spectrometry proteomics data have been deposited to the ProteomeXchange Consortium via the PRIDE partner repository with the dataset identifier PXD037364.

## Abbreviations

AMIPROM: Adapted method to isolate proteins from mucin
CRC: Colorectal cancer
CRS: Cytoreductive surgery
DIA: Data-independent acquisition
GO: Gene Ontology
HG-PMP: High-grade PMP
HIPEC: Hyperthermic intraperitoneal chemotherapy
IGs: Immunoglobulins
LG-PMP: Low-grade PMP
MS: Mass spectrometry
PLS-DA: Partial least squares-discriminant analysis
PMP: Pseudomyxoma peritonei
PPI: Protein–protein interaction
PSOGI: Peritoneal Surface Oncology Group International
SWAH-MS: Sequential window acquisition of all theoretical mass spectra

## References

[1] Sommariva A, Tonello M, Rigotto G, Lazzari N, Pilati P, Calabrò ML (2021). Novel Perspectives in Pseudomyxoma Peritonei Treatment. Cancers (Basel).

[2] Arjona-Sánchez Á, Muñoz-Casares FC, Rufián-Peña S, Díaz-Nieto R, Casado-Adam Á, Rubio-Pérez MJ (2011). Pseudomyxoma peritonei treated by cytoreductive surgery and hyperthermic intraperitoneal chemotherapy: results from a single centre. Clin Transl Oncol..

[3] Delhorme J-B, Severac F, Averous G, Glehen O, Passot G, Bakrin N (2018). Cytoreductive surgery and hyperthermic intraperitoneal chemotherapy for pseudomyxoma peritonei of appendicular and extra-appendicular origin. Br J Surg..

[4] Arjona-Sánchez Á, Muñoz-Casares FC, Casado-Adam Á, Sánchez-Hidalgo JM, Teran MDA, Orti-Rodriguez R (2013). Outcome of Patients with Aggressive Pseudomyxoma Peritonei Treated by Cytoreductive Surgery and Intraperitoneal Chemotherapy. World J Surg..

[5] Baratti D, Kusamura S, Milione M, Bruno F, Guaglio M, Deraco M (2017). Validation of the Recent PSOGI Pathological Classification of Pseudomyxoma Peritonei in a Single-Center Series of 265 Patients Treated by Cytoreductive Surgery and Hyperthermic Intraperitoneal Chemotherapy. Ann Surg Oncol..

[6] Carr NJ, Cecil TD, Mohamed F, Sobin LH, Sugarbaker PH, González-Moreno S (2016). A Consensus for Classification and Pathologic Reporting of Pseudomyxoma Peritonei and Associated Appendiceal Neoplasia: The Results of the Peritoneal Surface Oncology Group International (PSOGI) Modified Delphi Process. Am J Surg Pathol.

[7] Rufián-Andujar B, Valenzuela-Molina F, Rufián-Peña S, Casado-Adam Á, Sánchez-Hidalgo JM, Rodríguez-Ortiz L (2021). From the Ronnett to the PSOGI Classification System for Pseudomyxoma Peritonei: A Validation Study. Ann Surg Oncol [Internet]..

[8] Arjona-Sánchez Á, Martínez-López A, Valenzuela-Molina F, Rufián-Andujar B, Rufián-Peña S, Casado-Adam Á, et al. A Proposal for Modification of the PSOGI Classification According to the Ki-67 Proliferation Index in Pseudomyxoma Peritonei. Ann Surg Oncol. 2021;29:1–11. 10.1245/s10434-021-10372-9 34215955

[9] Arjona-Sanchez A, Martinez-López A, Moreno-Montilla MT, Mulsow J, Lozano-Lominchar P, Martínez-Torres B, et al. External multicentre validation of pseudomyxoma peritonei PSOGI-Ki67 classification. Eur J Surg Oncol. 2023; 49(8):1481-1488 10.1016/j.ejso.2023.03.206 36935222

[10] Lund-Andersen C, Torgunrud A, Fleten KG, Flatmark K (2020). Omics analyses in peritoneal metastasis—utility in the management of peritoneal metastases from colorectal cancer and pseudomyxoma peritonei: a narrative review. J Gastrointest Oncol..

[11] Pillai K, Akhter J, Mekkawy A, Chua TC, Morris DL (2017). Physical and chemical characteristics of mucin secreted by pseudomyxoma peritonei (PMP). Int J Med Sci..

[12] Amini A, Masoumi-Moghaddam S, Ehteda A, Morris DL (2014). Secreted mucins in pseudomyxoma peritonei: pathophysiological significance and potential therapeutic prospects. Orphanet J Rare Dis..

[13] Gum JR (1992). Mucin Genes and the Proteins They Encode: Structure, Diversity, and Regulation. Am J Resp Cell Mol..

[14] Hollingsworth MA, Swanson BJ (2004). Mucins in cancer: protection and control of the cell surface. Nat Rev Cancer..

[15] Mall AS, Chirwa N, Govender D, Lotz Z, Tyler M, Rodrigues J (2007). MUC2, MUC5AC and MUC5B in the mucus of a patient with pseudomyxoma peritonei: biochemical and immunohistochemical study. Pathol Int..

[16] Meldrum OW, Yakubov GE, Bonilla MR, Deshmukh O, McGuckin MA, Gidley MJ (2018). Mucin gel assembly is controlled by a collective action of non-mucin proteins, disulfide bridges, Ca2+-mediated links, and hydrogen bonding. Sci Rep-uk..

[17] Dayal S, Taflampas P, Riss S, Chandrakumaran K, Cecil TD, Mohamed F (2013). Complete cytoreduction for pseudomyxoma peritonei is optimal but maximal tumor debulking may be beneficial in patients in whom complete tumor removal cannot be achieved. Dis Colon Rectum..

[18] Smeenk RM, van Velthuysen MLF, Verwaal VJ, Zoetmulder FAN (2008). Appendiceal neoplasms and pseudomyxoma peritonei: a population based study. Eur J Surg Oncol..

[19] Patrick-Brown TDJH, Carr NJ, Swanson DM, Larsen S, Mohamed F, Flatmark K (2021). Estimating the Prevalence of Pseudomyxoma Peritonei in Europe Using a Novel Statistical Method. Ann Surg Oncol..

[20] Shilov IV, Seymour SL, Patel AA, Loboda A, Tang WH, Keating SP (2007). The Paragon Algorithm, a Next Generation Search Engine That Uses Sequence Temperature Values and Feature Probabilities to Identify Peptides from Tandem Mass Spectra*. Mol Cell Proteomics..

[21] Perez-Riverol Y, Bai J, Bandla C, García-Seisdedos D, Hewapathirana S, Kamatchinathan S (2021). The PRIDE database resources in 2022: a hub for mass spectrometry-based proteomics evidences. Nucleic Acids Res..

[22] Zhou Y, Zhou B, Pache L, Chang M, Khodabakhshi AH, Tanaseichuk O (2019). Metascape provides a biologist-oriented resource for the analysis of systems-level datasets. Nat Commun..

[23] Bader GD, Hogue CW (2003). An automated method for finding molecular complexes in large protein interaction networks. BMC Bioinform..

[24] Singhi AD, Davison JM, Choudry HA, Pingpank JF, Ahrendt SA, Holtzman MP (2014). GNAS is frequently mutated in both low-grade and high-grade disseminated appendiceal mucinous neoplasms but does not affect survival. Hum Pathol..

[25] Shetty S, Thomas P, Ramanan B, Sharma P, Govindarajan V, Loggie B (2013). Kras mutations and p53 overexpression in pseudomyxoma peritonei: association with phenotype and prognosis. J Surg Res..

[26] Pietrantonio F, Perrone F, Mennitto A, Gleeson EM, Milione M, Tamborini E (2016). Toward the molecular dissection of peritoneal pseudomyxoma. Ann Oncol..

[27] Luo C, Cen S, Ding G, Wu W (2019). Mucinous colorectal adenocarcinoma: clinical pathology and treatment options. Cancer Commun..

[28] Pillai K, Akhter J, Morris DL (2017). Assessment of a novel mucolytic solution for dissolving mucus in pseudomyxoma peritonei: an ex vivo and in vitro study. Pleura Peritoneum..

[29] Pavlova NN, Thompson CB (2016). The Emerging Hallmarks of Cancer Metabolism. Cell Metab..

[30] Kos J (2022). Proteases: Role and Function in Cancer. Int J Mol Sci..

[31] Fane M, Weeraratna AT (2020). How the ageing microenvironment influences tumour progression. Nat Rev Cancer..

[32] Williams SJ, Wreschner DH, Tran M, Eyre HJ, Sutherland GR, McGuckin MA (2001). MUC13, a Novel Human Cell Surface Mucin Expressed by Epithelial and Hemopoietic Cells*. J Biol Chem.

[33] Malik S, Sikander M, Wahid M, et al. Deciphering cellular and molecular mechanism of MUC13 mucin involved in cancer cell plasticity and drug resistance. Cancer Metastasis Rev. 2024. 10.1007/s10555-024-10177-8. 10.1007/s10555-024-10177-8 PMC13394004 38498072

[34] Filippou PS, Ren AH, Korbakis D, Dimitrakopoulos L, Soosaipillai A, Barak V (2018). Exploring the potential of mucin 13 (MUC13) as a biomarker for carcinomas and other diseases. Clin Chem Lab Med (CCLM)..

