# Supplementary material for: Breaking the Mucin Barrier: A New Affinity Chromatography-Mass Spectrometry Approach to Unveil Potential Cell Markers and Pathways Altered in Pseudomyxoma Peritonei
Source: Biol Proced Online. 2024 May 15;26:13. doi: 10.1186/s12575-024-00239-0 (PMC11094946; doi:10.1186/s12575-024-00239-0)
Supplement: Supplementary file 1 — Supplementary Material 1. [file 12575_2024_239_MOESM1_ESM.docx]

**Supplementary Material**

**Methods**

*Protein identification in the depleted extracts using Liquid Chromatography coupled to Mass Spectrometry (LC-MS).*

The extracts (elution peaks) captured on the HiTrap Con A 4B column and the HiTrap Albumin and IgG Depletion column were loaded onto a 10% acrylamide gel and a electrophoresis was run. The main bands were then excised and sent to the IMIBIC Mass Spectrometry and Molecular Imaging Service (IMSMI) of the Maimónides Biomedical Research Institute of Cordoba for protein identification analysis. Briefly, and prior to analysis, the sample preparation process included digestion of the gel bands, followed by total protein amount quantification for each sample by microfluorimetry (Qubit™ Protein Assay; Thermo Fisher Scientific). A total amount of 20 μl of the recovered diluted peptides was then loaded onto Evotips (Evosep). Pierce™ Hela Tryptic Digest Standard (Thermo Fisher Scientific) was also prepared and loaded (200 ng) onto Evotips for quality control and system equilibration. Next, purified peptides were separated using the predefined 30 SPD method (44-min gradient time) on an Evosep One LC system (Evosep) ^1^. A ZDV emitter (Bruker Daltonics) was placed inside a nanoelectrospray source (CaptiveSpray source, Bruker Daltonics). The emitter was connected to a 15-cm × 150-μm reverse-phase column packed with 1.9-μm C18 beads (1893471). The column was heated to 60ºC in an oven compartment (Bruker Daltonics). Mobile phases were water and acetonitrile, buffered with 0.1% formic acid (LC-MS grade, Fisher Scientific). Liquid chromatography was coupled online to a TIMS Q-TOF instrument (timsTOF Pro, Bruker Daltonics) using the Data Dependent Acquisition-Parallel Accumulation SErial Fragmentation (DDA-PASEF) method ^2,3^ via a CaptiveSpray nano-electrospray ion source. For the acquisition mode, the ion mobility dimension was calibrated with three Agilent ESI-L Tuning Mix ions (m/z, 1/K0: 622.0289 Th, 0.9848 Vs cm−2; 922.0097 Th, 1.1895 Vs cm−2; 1221.9906 Th, 1.3820 Vs cm−2). Furthermore, the collision energy was linearly reduced from 59 eV at 1/K0 = 1.6 Vs cm−2 to 20 eV at 1/K0 = 0.6 Vs cm−2. For the DDA-PASEF method the “long gradient method” (1.1s cycle time) was used and the accumulation and ramp times were set to 100 ms. Singly charged precursors were excluded from fragmentation using a polygon filter in the (m/z, 1/K0) plane. In addition, all precursors reaching the target value of 20,000 were excluded for 0.4 min. Precursors were isolated using a Q window of 2 Da for m/z <700 and 3 Da for m/z >800.

Data analysis and protein identification were performed on samples acquired in DDA-PASEF mode. This was done using the computational platform FragPipe (version 20.0), which includes MSFragger ^4,5^. Peptide identification from tandem mass spectra (MS/MS) was performed using the MSFragger search engine, with either raw (.d) files as input. The Human Reference Proteome (https://www.uniprot.org/taxonomy/9606) version fasta file was downloaded from Uniprot (https://uniprot.org) and used as the search database. Reversed protein sequences were appended to the original databases as decoys. Additionally, for each analysis, the MS/MS search results were further processed using the Philosopher toolkit ^6^. Each identified peptide was either assigned as a unique peptide to a particular protein (or protein group containing indistinguishable proteins) or assigned as a razor peptide to a single protein (protein group) that had the most peptide evidence. The protein groups with the probability of the best peptide used such as the protein-level score ^7^, were filtered to 1% protein-level FDR using the picked FDR strategy ^8^, allowing for unique and razor peptides. The final reports were then generated and filtered at each level (PSM, ion, peptide, and protein) using the 2D FDR approach ^9^ (1% protein FDR plus 1% PSM/ion/peptide-level FDR for each corresponding PSM.tsv, ion.tsv, and peptide.tsv files).

**References**

1. Bache N, Geyer PE, Bekker-Jensen DB, Hoerning O, Falkenby L, Treit PV, et al. A Novel LC System Embeds Analytes in Pre-formed Gradients for Rapid, Ultra-robust Proteomics*. Mol Cell Proteom. 2018;17(11):2284–96.

2. Meier F, Brunner AD, Frank M, Ha A, Bludau I, Voytik E, et al. diaPASEF: parallel accumulation–serial fragmentation combined with data-independent acquisition. Nat Methods. 2020;17(12):1229–36.

3. Meier F, Brunner AD, Koch S, Koch H, Lubeck M, Krause M, et al. Online Parallel Accumulation–Serial Fragmentation (PASEF) with a Novel Trapped Ion Mobility Mass Spectrometer*. Mol Cell Proteom. 2018;17(12):i–2545.

4. Kong AT, Leprevost FV, Avtonomov DM, Mellacheruvu D, Nesvizhskii AI. MSFragger: ultrafast and comprehensive peptide identification in mass spectrometry–based proteomics. Nat Methods. 2017;14(5):513–20.

5. Yu F, Haynes SE, Teo GC, Avtonomov DM, Polasky DA, Nesvizhskii AI. Fast Quantitative Analysis of timsTOF PASEF Data with MSFragger and IonQuant. Mol Cell Proteom. 2020;19(9):1575–85.

6. Leprevost F da V, Haynes SE, Avtonomov DM, Chang HY, Shanmugam AK, Mellacheruvu D, et al. Philosopher: a versatile toolkit for shotgun proteomics data analysis. Nat Methods. 2020;17(9):869–70.

7. Nesvizhskii AI. A survey of computational methods and error rate estimation procedures for peptide and protein identification in shotgun proteomics. J Proteom. 2010;73(11):2092–123.

8. Savitski MM, Wilhelm M, Hahne H, Kuster B, Bantscheff M. A Scalable Approach for Protein False Discovery Rate Estimation in Large Proteomic Data Sets[S]. Mol Cell Proteom. 2015;14(9):2394–404.

9. Bern MW, Kil YJ. Two-Dimensional Target Decoy Strategy for Shotgun Proteomics. J Proteome Res. 2011;10(12):5296–301.

**Figure S1:** Representative image of the electrophoresis in a 10% acrylamide gel of the elution peaks that were captured by the two columns in the liquid chromatography. “First Column” (FC) corresponds to the elution peak of the HiTrap Con A 4B column and “second column” (SC) corresponds to the elution peak of the HiTrap Albumin and IgG Depletion column. The bands analysed by mass spectrometry are shown in red. MW: molecular weight.

**Table S1.** Total number of proteins and localizations identified in the fractions captured by the HiTrap Con A 4B column and by the HiTrap Albumin and IgG Depletion column.

| *HiTrap Con A 4B column (First column)* | | | |
| --- | --- | --- | --- |
| Total protein number | **Secreted proteins** | **Cell membrane proteins** | **Cytosolic proteins** |
| 85 | 82 | 15* | 7 |
| *HiTrap Albumin and IgG Depletion column (Second column)* | | | |
| Total protein number | **Secreted proteins** | **Cell membrane proteins** | **Cytosolic proteins** |
| 16 | 16 | 7* | 0 |

* 10 out of 15 (66.7%) of the identified cell membrane proteins captured in the first column and all (100%) of the identified cell membrane proteins captured in the second column were immunoglobulins. It is important to note that immunoglobulins were one of the targets to be depleted.

**Table S2**: Functional enrichment analysis derived from the clusters obtained from the protein-protein interaction networks generated using MCODE algorithm and Metascape database for two protein lists (SM vs. CTRL and HM vs. CTRL). All the proteins identified in each cluster and the top three best p-value terms are shown in the table.

| **Soft and Hard Mucin vs. Control** | | | | | |
| --- | --- | --- | --- | --- | --- |
| **Cluster** | **Gene Symbol** | **Category** | **Term name** | **Description** | **Log10(p-value)** |
| C1 | G3BP1, DENR, MIF, ENO2, ADH1B, MSN, PTMA, TAGLN, ADH7, PRKCSH, PGK1, HSPA1A, FUS, FGG, JUP, FGA, HSP90AB1, NPM1, ANP32A, FKBP1A, PPIA, PHYKPL, XRCC6, TUBB4B, ARHGDIB, ACO2, FGB, FABP4, ACAT2, TMPO, MYH14, IDH1, STX7, ECI1, PRDX3, EIF1, UBA52, VCL, FABP5, DSP, AK2, FKBP2 | GO CC | GO:0034774 | Secretory granule lumen | -13.9 |
|  |  | GO CC | GO:0060205 | Cytoplasmic vesicle lumen | -13.8 |
|  |  | GO CC | GO:0031983 | Vesicle lumen | -13.8 |
| C2 | NIT2, PTGES3, RAN, ITIH3, CLEC3B, LSM7, RARRES2, PRDX1, PRDX4, FAM3C, PDE6D, LGALS3BP, ORM1, SERPINA1, GPX4, CALM1, EWSR1, ARPC1B, RNH1, STMN1, DUT, NUTF2, DBI, HNRNPC, HNRNPA2B1, CNDP2, PLPBP, RAP1B, ALB, GLUD1, CLTB, GSTO1, HNRNPA1 | Reactome | R-HSA-114608 | Platelet degranulation | -13.9 |
|  |  | Reactome | R-HSA-76005 | Response to elevated platelet cytosolic Ca2+ | -13.8 |
|  |  | Reactome | R-HSA-76002 | Platelet activation, signaling and aggregation | -12.8 |
| C3 | ATP5F1B, GNAI2, ANXA6, MDH2, PPIB, PRDX5, ALDH2, GOT1, HSPA8, LAP3, DLST, ENO1, HSPA5, HSPA2, LDHA, EEF2, ALDOA, HSPD1, LDHB, AKR1A1, NCL, EIF4B, VCP, CNN1, SOD2, DSTN, UBE2L3 | WikiPathways | WP534 | Glycolysis and gluconeogenesis | -11.7 |
|  |  | KEGG | hsa00010 | Glycolysis / Gluconeogenesis | -10.6 |
|  |  | GO BP | GO:0006163 | Purine nucleotide metabolic process | -9.8 |
| C4 | COL1A1, CD55, HCLS1, COL12A1, CRKL, ARPC4, UBE2I, MARCKS, SPTBN1, VPS26A, UBE2V2, ANXA2, COL15A1, BASP1, CORO1C, COL18A1, ANK1, RAC1, HSPG2, UBE2N, MARCKSL1, EPB41L2, GRB2, RRAS, CD59, CLTA | GO BP | GO:0097435 | Supramolecular fiber organization | -10.5 |
|  |  | KEGG | hsa05205 | Proteoglycans in cancer | -9.5 |
|  |  | GO BP | GO:0030036 | Actin cytoskeleton organization | -9.4 |
| C5 | APOM, CAP1, ATP6V1A, CFL1, PGM1, PGD, APOA4, RPL5, SOD1, FN1, PPA1, TPI1, APOH, APOE, RPSA, AHCY, TEP1, CLTC, PPA2, PLTP, APOA2, TARS1, NME1, APOA1, CLU | GO CC | GO:0034364 | High-density lipoprotein particle | -19.0 |
|  |  | GP CC | GO:1990777 | Lipoprotein particle | -17.7 |
|  |  | GO CC | GO:0034358 | Plasma lipoprotein particle | -17.7 |
| C6 | PSMD9, PSME1, RACK1, RPL10A, PSMB1, RPS3, PSMA2, RPS25, EEF1B2, SNCA, RPL18, SRP9, PSMA6, RPL12, PSMA1, PSMB6, PSMB8, SRP14, RPS28, SERBP1, HNRNPD | Reactome | R-HSA-9010553 | Regulation of expression of SLITs and ROBOs | -26.6 |
|  |  | Reactome | R-HSA-376176 | Signaling by ROBO receptors | -25.1 |
|  |  | Reactome | R-HSA-71291 | Metabolism of amino acids and derivatives | -21.8 |
| C7 | LBR, AHNAK, TMOD3, ARF6, CALD1, YWHAH, COTL1, PFN1, CHMP2A, EZR, AKAP12, PALM, SH3BGRL, RHOA, PDCD6IP | GO CC | GO:0005938 | Cell cortex | -10.2 |
|  |  | GO CC | GO:0005925 | Focal adhesionn | -9.3 |
|  |  | GO CC | GO:0030055 | Cell-substrate junction | -9.2 |
| C8 | PPP2CA, YWHAE, PPP1CB | KEGG | hsa04114 | Oocyte meiosis | -7.1 |
|  |  | KEGG | hsa04390 | Hippo signaling pathway | -6.9 |
|  |  | Reactome | R-HSA-69275 | G2/M Transition | -6.6 |
| C9 | IGF2, IGFBP3, MMP2 | Reactome | R-HSA-381426 | Regulation of Insulin-like Growth Factor (IGF) transport and uptake by Insulin-like Growth Factor Binding Proteins (IGFBPs) | -7.2 |
|  |  | GO BP | GO:0001503 | Ossification | -6.1 |

**Table S3**: Functional enrichment analysis derived from the clusters obtained from the protein-protein interaction networks generated using MCODE algorithm and Metascape database for three protein lists in soft mucin (SM) tissues (LG vs. CTRL, HG vs. CTRL and LG vs. HG). All the proteins identified in each cluster and the top three best p-value terms are shown in the table. ND: not detected. No functional enrichment derived from that cluster.

| **Low grade and High grade SM vs Control** | | | | | |
| --- | --- | --- | --- | --- | --- |
| **Cluster** | **Gene Symbol** | **Category** | **Term name** | **Description** | **Log10(p-value)** |
| C1 | FKBP1A, BLMH, SOD2, RRAS, EIF4B, ACP1, MYH14, LDHA, NPM1, HSP90AB1, TMPO, CNN1, TAGLN2, EWSR1, HSPA2, MARCKSL1, DSC1, UBA52, AKAP12, ENO2, SERPINB12, LSM7, CALM1, MAGOHB, DENR, XRCC6, TAGLN, RBMX, STX7, HNRNPA2B1, MDH2, AP1B1, PFN1, CDC42 | WikiPathways | WP3888 | VEGFA VEGFR2 signaling | -6.4 |
|  |  | Reactome | R-HSA-194315 | Signaling by Rho GTPases | -6.1 |
|  |  | Reactome | R-HSA-9716542 | Signaling by Rho GTPases, Miro GTPases and RHOBTB3 | -6.0 |
| C2 | PPA2, PRDX5, ACTR2, PPA1, FKBP2, MSN, ARF4, ENO1, SERBP1, PTMS, CCT8, RPL10A, AHCY, LAP3, PGM1, LDHB, CHMP4B, RACK1, GPX4, RAB11B, ATP5F1B, PA2G4, FABP5, ALDH2, EEF2, TARS1, PPIB, ANXA6 | GO MF | GO:0045296 | cadherin binding | - 7.8 |
|  |  | GO MF | GO:0050839 | cell adhesion molecule binding | -7.5 |
|  |  | KEGG | hsa00010 | Glycolysis / Gluconeogenesis | - 6.4 |
| C3 | BASP1, TPI1, MIF, NME1, EIF1, ANXA2, DLST, GSTO1, AKR1A1, GOT1, GRB2, FUS, PTMA, PPIA, STMN1, LSM4, FABP4, AK2, LSM2, ECI1, HNRNPC, DUT, CFL1, ARF1, NUTF2 | Reactome | R-HSA-8950505 | Gene and protein expression by JAK-STAT signaling after Interleukin-12 stimulation | - 12.3 |
|  |  | Reactome | R-HSA-9020591 | Interleukin-12 signaling | - 11.8 |
|  |  | Reactome | R-HSA-447115 | Interleukin-12 family signaling | - 11.2 |
| C4 | PSMA1, RPL18, PSMB6, PSMB1, RPL5, SRP9, PSMB8, PSMD9, SNCA, HNRNPD, PSMA2 | Reactome | R-HSA-450408 | AUF1 (hnRNP D0) binds and destabilizes mRNA | - 16.8 |
|  |  | Reactome | R-HSA-9010553 | Regulation of expression of SLITs and ROBOs | - 15.8 |
|  |  | Reactome | R-HSA-450531 | Regulation of mRNA stability by proteins that bind AU-rich elements | - 15.3 |
| C5 | PHYKPL, HDGF, PDCD6IP, SUB1, HSPA8, PEPD | ND | | | |
| C6 | EZR, RAC1, ARF6 | GO BP | GO:0030865 | cortical cytoskeleton organization | - 8.4 |
|  |  | GO CC | GO:0001726 | ruffle | - 6.7 |
|  |  | KEGG | hsa05130 | Pathogenic Escherichia coli infection | - 6.6 |
| C7 | VCL, CALD1, MYL6 | Reactome | R-HSA-445355 | Smooth Muscle Contraction | - 8.5 |
|  |  | Reactome | R-HSA-397014 | Muscle contraction | - 6.5 |
|  |  | GO CC | GO:0015629 | actin cytoskeleton | - 5.3 |

| **Low grade and High grade HM vs Control** | | | | | |  |  |  |
| --- | --- | --- | --- | --- | --- | --- | --- | --- |
| **Cluster** | **Gene Symbol** | **Category** | **Term name** | **Description** | **Log10(p-value)** |  |  |  |
| C1 | ENO2, ATP6V1A, PGM1, PSMD9, GDI2, PSMA4, PSMB1, HK1, PRDX5, PSMA2, DSTN, SNCA, UBA1, PGAM1, PSMB4, GPI, PSMA6, DLST, ACO2, EIF4E, BPGM, RAB5C, PSMA1, PSMB6, GALT, ALDH1A2, PSMB5 | CORUM | CORUM:191 | 20S proteasome | -21.4 |  |  |  |
|  |  | CORUM | CORUM:194 | PA28gamma-20S proteasome | -21.1 |  |  |  |
|  |  | CORUM | CORUM:192 | PA28-20S proteasome | -20.8 |  |  |  |
| C2 | COL1A1, COL1A2, COL12A1, COL6A3, PPIB, ARPC3, TUBB, ARPC4, SOD1, ALDH2, PRDX2, COL14A1, COL15A1, PPIE, ARPC1B, EIF5A, GOT2, ARPC2, COL18A1, NEDD8, PRDX3, DLD, COL4A1, CALML3 | Reactome | R-HSA-1650814 | Collagen biosynthesis and modifying enzymes | -18.0 |  |  |  |
|  |  | Canonical Pathways | M3005 | NABA COLLAGENS | -17.1 |  |  |  |
|  |  | Reactome | R-HSA-8948216 | Collagen chain trimerization | -17.1 |  |  |  |
| C3 | HCLS1, HNRNPL, SNRPF, YBX1, SNRPD2, CKB, SNRPD3, ACTR2, PGK1, LDHB, ACTR3, HNRNPC, LSM2, NME1, FABP5, FKBP1A | WikiPathways | WP411 | mRNA processing | -12.7 |  |  |  |
|  |  | Reactome | R-HSA-72163 | mRNA Splicing - Major Pathway | -11.2 |  |  |  |
|  |  | Reactome | R-HSA-72172 | mRNA Splicing | -11.1 |  |  |  |
| C4 | C3, DCD, AKAP12, C4B, HSPA8, DSG1, SERPINB12, CFD, YWHAH, MAPK1, C4A | Reactome | R-HSA-166663 | Initial triggering of complement | -10.1 |  |  |  |
|  |  | WikiPathways | WP545 | Complement activation | -10.1 |  |  |  |
|  |  | KEGG | hsa05150 | Staphylococcus aureus infection | -9.9 |  |  |  |
| C5 | CD55, ARF4, MDH2, SPTA1, RPS21, ALDOA, PCNA, ANXA6, PLEC, RPS5, ANK1 | Reactome | R-HSA-6807878 | COPI-mediated anterograde transport | -7.4 |  |  |  |
|  |  | Reactome | R-HSA-199977 | ER to Golgi Anterograde Transport | -6.7 |  |  |  |
|  |  | Reactome | R-HSA-948021 | Transport to the Golgi and subsequent modification | -6.4 |  |  |  |
| C6 | NIT2, ADH1B, CP, MDH1, YWHAQ, YWHAG, S100A11, ORM1, SERPINA3, IDH2, SERPINA1 | GO CC | GO:0034774 | secretory granule lumen | -7.2 |  |  |  |
|  |  | GO CC | GO:0060205 | cytoplasmic vesicle lumen | -7.2 |  |  |  |
|  |  | GO CC | GO:0031983 | vesicle lumen | -7.2 |  |  |  |
| C7 | APOA2, APOA4, LPA, APOH, APOL1, CLU, PLTP | GO CC | GO:0034358 | plasma lipoprotein particle | -20.7 |  |  |  |
|  |  | GO CC | GO:1990777 | lipoprotein particle | -20.7 |  |  |  |
|  |  | GO CC | GO:0032994 | protein-lipid complex | -20.5 |  |  |  |
| C8 | | KRAS, RHOA, MSN, GNB1 | Canonical Pathways | M41 | PID ER NONGENOMIC PATHWAY | -11.6 |  |  |
|  |  |  | WikiPathways | WP35 | G protein signaling pathways | -7.0 |  |  |
|  |  |  | Reactome | R-HSA-3858494 | Beta-catenin independent WNT signaling | -6.4 |  |  |
| C9 | | CDC42, LBR, TMPO | Reactome | R-HSA-9013408 | RHOG GTPase cycle | -7.9 |  |  |
|  |  |  | Reactome | R-HSA-9013404 | RAC2 GTPase cycle | -7.6 |  |  |
|  |  |  | Reactome | R-HSA-9013423 | RAC3 GTPase cycle | -7.5 |  |  |
| C10 | | | PRDX6, GLO1, HSPB1 | ND | | | |  |
| C11 | | | FBP1, ALB, KRT6A | ND | | | |  |
| C12 | | | | VTN, F2  KNG1 | GO BP | GO:0030195 | negative regulation of blood coagulation | -8.5 |
|  |  |  |  |  | GO BP | GO:1900047 | negative regulation of hemostasis | -8.5 |
|  |  |  |  |  | GO BP | GO:0050819 | negative regulation of coagulation | -8.4 |

**Table S4**: Functional enrichment analysis derived from the clusters obtained from the protein-protein interaction networks generated using MCODE algorithm and Metascape database for three protein lists in hard mucin (HM) tissues (LG vs. CTRL, HG vs. CTRL and LG vs. HG). All the proteins identified in each cluster and the top three best p-value terms are shown in the table. ND: not detected. No functional enrichment derived from these clusters.
